# Establishing self-concordant goals: a longitudinal study on the influence of ego identity on goal self-concordance

**DOI:** 10.3389/fpsyg.2024.1382345

**Published:** 2024-07-10

**Authors:** Lu Cao, Jianhong Ma

**Affiliations:** Department of Psychology and Behavioral Sciences, Zhejiang University, Hangzhou, China

**Keywords:** goal self-concordance, ego identity, longitudinal study, cross-lagged analysis, goal

## Abstract

**Introduction:**

Self-concordant goals are those that align closely with an individual’s intrinsic interests and values, driving autonomous motivation, and resulting in higher satisfaction and goal achievement. The relevance of studying the link between ego identity and goal self-concordance lies in understanding how a well-defined ego identity can foster the pursuit of authentic and fulfilling goals.

**Objectives:**

This study investigates the relationship between ego identity and goal self-concordance, focusing on how individuals’ sense of identity influences their alignment with personal goals.

**Methods:**

Data were gathered through a cross-sectional analysis of 292 students and a longitudinal follow-up with 98 participants over two intervals.

**Results:**

Results confirmed significant correlations between ego identity status and various dimensions of goal self-concordance. Notably, different ego identity statuses exhibited distinct levels of goal self-concordance, ranked from highest to lowest as follows: identity achievement, achievement-foreclosure intermediate, moratorium, foreclosure, moratorium-diffusion intermediate, and diffusion. Cross-lagged analysis revealed a causal relationship where ego identity influences goal self-concordance, providing insights into the cognitive and behavioral processes involved in goal setting.

**Discussion:**

This study extends the implications of the goal self-concordance model and reveals a causal relationship between ego identity status and goal self-concordance. From a practical perspective, our findings suggest that educational and developmental interventions aimed at supporting identity development and commitment could lead to more self-concordant goal setting. Such programs could ultimately promote greater well-being and personal growth among students.

## 1 Introduction

Individuals often regard happiness as their ultimate goal. Research, including Sheldon’s (2005) study, indicates that people are innately inclined to seek happiness. However, what we desire, or believe we desire, may not invariably lead to the anticipated happiness. Commonly, people associate increased wealth, attractiveness, or popularity with happiness; however, these achievements might only fuel further desires (Sheldon et al., 2010).

Sheldon and Elliot (1999), building on the self-determination theory, proposed the concept of goal self-concordance to model the relationship between goals and happiness. This concept pertains to how closely an individual’s goals align with their intrinsic interests and values. They argue that these intrinsic elements form part of an individual’s “self,” motivating and inspiring action. When goals are highly concordant with the self, they reflect the individual’s genuine interests, values, and identity, driven by strong autonomous motivation. By contrast, low concordance suggests that goals are more influenced by external pressures or negative emotions, indicative of controlled motivation. Self-concordant goals align with a person’s true interests, passions, core values, and beliefs, whereas incongruent goals are pursued out of obligation. Research conducted over several years, including studies by Sheldon and Elliot (1999), Sheldon and Houser-Marko (2001), and Sheldon et al. (2004), has shown that pursuing highly self-concordant goals leads to increased positive emotions and satisfaction, enhancing the individual’s motivation to exert effort, and facilitating smoother goal achievement. These achievements are intricately linked to fulfilling everyday needs such as autonomy, competence, and interpersonal relationships. Over time, fulfilling these needs tends to boost overall happiness (Smith et al., 2011; Sheldon et al., 2019). Thus, the achievement of self-concordant goals positively leads to fulfillment of needs, a positive mindset, and successful goal attainment.

However, despite these findings affirming the correlation between goals with high self-concordance and greater happiness, a lingering question persists: why, despite our free will, do we sometimes choose goals that are discordant with our self and potentially detrimental to our happiness?

Humans, as social animals, are frequently subject to the influence of societal pressures. These pressures can divert us from pursuing our true desires, leading us toward activities we perceive as obligatory (Werner and Milyavskaya, 2018). Consequently, selecting self-concordant goals may comprise a “difficult skill,” necessitating precise self-awareness and resilience against societal forces that may inadvertently guide individuals in unsuitable directions (Sheldon and Houser-Marko, 2001). The question then arises: How can one develop this “difficult skill”?

In his theory of psychosocial development, Erikson (1959, 1968) articulated the concept of “ego identity.” He proposed that ego identity encompassed an individual’s beliefs and thoughts concerning their own identity in various dimensions. It is a personal, subjective experience of consistency and continuity within oneself over time—past, present, and future—and extends to how this consistency is perceived by others. This construct signifies an individual’s integration of the self within a particular context. The formation of ego identity is thus a pivotal developmental task in one’s life.

Expanding on Erikson’s framework, Marcia (1966, 1987) identified four identity-development statuses in adolescence: identity achievement, identity moratorium, identity foreclosure, and identity diffusion. Kato (1983) developed the Ego Identity Status Scale based on Marcia’s theory of ego identity statuses. This scale comprises three subscales: present commitment, past crisis, and future commitment pursuit. It assigns one of six identity statuses using a designated flowchart, which incorporates the subtotals of the three subscales as inputs. The statuses are ranked from low to high developmental levels: identity diffusion, diffusion-moratorium intermediate, moratorium, foreclosure, foreclosure-achievement intermediate, and achievement. Zhang (2000) adapted this scale to the Chinese context and designed a flowchart for assessing various ego identity status, popularizing the Chinese version among Chinese researchers.

Numerous studies have established that ego identity closely relates to the formation and development of personal goals. Erikson (1968) posited that individuals with well-established ego identities tended to strengthen their self-awareness, foster positive and healthy relationships, demonstrate care and societal contribution, and have a clear life purpose, all of which were crucial for individual growth. Schwartz et al. (2005) characterized ego identity as a significant anchor in the sea of life’s possibilities, providing guidance and motivation. Zhang (2021) discovered a positive correlation between the three dimensions of the scale—present commitment, past crisis, and future commitment pursuit—and a sense of life purpose. Thus, it is plausible that the level of ego identity status influences goal selection. Therefore, this study proposes the following hypothesis:

*Hypothesis 1: Individuals with a higher level of ego identity status will demonstrate higher levels of goal self-concordance.*

*Hypothesis 2: There will be significant differences in goal self-concordance among different ego identity status.*

To verify this hypothesis, a cross-sectional analysis was conducted, which yielded preliminary insights into this relationship; however, the analysis’ inherent limitations, especially regarding causality, underscore the necessity for more comprehensive research. This is the context in which longitudinal studies gain importance. Although the interaction mechanisms between goal self-concordance and ego identity status remain underexplored in existing research, the connection between goals and identity has been extensively examined. Some scholars postulate that ego identity may precede the formation of life goals, while Schwartz et al. (2005) argued that ego identity laid the groundwork for individual goal formation. Burrow and Hill (2020) suggested that ego identity formed the psychological underpinning for developing life purpose. Successfully navigating ego identity crises enables individuals to develop new strengths and capabilities, thus fostering the exploration and achievement of broader goals. In a 5-year longitudinal study involving in-depth interviews with eight adolescents, Bronk (2012) concluded that the establishment of ego identity in adolescents was instrumental in developing a comprehensive, clear self-concept, which subsequently led to more defined goals and heightened commitment and effort.

Some researchers consider the relationship from a different perspective. Côté (1997) introduced the identity capital model theory, positing that identity capital was the psychological capacity that enabled individuals to successfully complete tasks and use their experiences. A stable ego identity can improve an individual’s ability to understand and navigate related challenges and opportunities, thereby facilitating task completion and the effective utilization of experiences. Furthermore, having clear goals is identified as a crucial source of identity capital. Kashdan and McKnight (2009) discovered that the interplay between goals and ego identity assisted in the efficient allocation of limited resources toward meaningful tasks. Benson et al. (2006) underscored the importance of having clear personal goals, deeming them key factors in adolescent development. Liu (2014) examined the relationship between intrinsic and extrinsic goals and ego identity status from the perspective of self-determination theory, finding that intrinsic goals positively impacted ego identity status, whereas extrinsic goals negatively impacted it. These studies collectively suggest that personal goals can significantly predict and shape ego identity.

Other researchers argue for a reciprocal effect between goals and ego identity. The pursuit of personal goals offers a route to self-direction and self-definition. Individuals actively shape their own development by selecting goals, formulating strategies for achieving them, and committing to the necessary efforts for realization. This dynamic aids in defining the roles they assume, constructing their unique narratives, and enhancing self-assessment. In turn, the evolution of ego identity influences an individual’s expectations regarding goal outcomes, the selection of goals, and methodologies for goal attainment. Thus, the relationship between ego identity and personal goals is characterized as a continuous, interactive process (Nurmi et al., 1994; Seginer, 2005).

Considering varied viewpoints, it becomes apparent that the interaction between goals and ego identity is complex and multifaceted. The causal relationship between goal self-concordance and identity status remains underexplored. Do the changes in ego identity status influence goal self-concordance, or does goal self-concordance affect ego identity status, or do they mutually influence each other? This study suggests that individuals with a well-defined ego identity are more likely to pursue goals that align with their intrinsic values and interests, thereby exhibiting higher goal self-concordance. Therefore, a cross-hysteresis model is designed in this study and the following hypotheses are proposed:

*Hypothesis 3: Ego identity status is the cause and goal self-concordance is the effect.*

## 2 Methods

### 2.1 Participants and procedure

This research focused on university students in China, encompassing two distinct surveys. All study participants provided informed consent, and the study design was approved by the appropriate ethics review board. The first survey, a cross-sectional design, sought to investigate the relationship between ego identity, goal self-concordance, and their respective dimensions. Distributed via an online questionnaire platform, 351 responses were received. Excluding surveys that failed attention checks (e.g., “Please select option 2 for this question”) and those with uniform responses across items, 292 valid responses were retained. The sample comprised 203 males (69.5%) and 89 females (30.5%), averaging 20.34 years of age (SD = 0.84). The participants were asked to define their most significant recent goal and were evaluated for goal self-concordance and ego identity status.

The second set of surveys, designed longitudinally, aimed to delineate the causal relationship between goal self-concordance and ego identity using a cross-lagged panel model. Initially, the participants identified their primary goal, undergoing assessment for goal self-concordance and ego identity status. A follow-up survey 3 months later repeated this process. The first survey yielded 139 responses, with 112 follow-ups in the second. Applying similar exclusion criteria, 98 valid paired responses were obtained for analysis, including 59 males (60.2%) and 39 females (39.8%).

SPSS 24.0 was used for descriptive statistics and correlational analyses, while Mplus 8.0 facilitated the cross-lagged analysis.

### 2.2 Sample size adequacy and power analysis

Ensuring adequate sample sizes is crucial to verify the reliability of our findings. We conducted a power analysis to determine the sample sizes required for detecting significant effects with adequate power.

For the cross-sectional analysis, we involved 292 students. We performed a power analysis using G*Power software, specifying a medium effect size (Cohen’s *d* = 0.30), an alpha level of 0.05, and a desired power of 0.80. The analysis indicated that a minimum sample size of approximately 143 participants was required to detect medium effects. Thus, our sample size of 292 exceeds this requirement, ensuring robust and reliable results.

In the longitudinal study, we collected data from 98 participants at two different time points. Despite the smaller sample size, we conducted a power analysis for repeated measures ANOVA, considering a medium effect size (Cohen’s *f* = 0.25), an alpha level of 0.05, and a desired power of 0.80. The power analysis suggested that at least 34 participants were needed to detect significant changes over time. Therefore, our sample size of 98 is adequate for detecting medium effects in longitudinal analysis and accounts for potential attrition over time.

These analyses confirm that the sample sizes used in both the cross-sectional and longitudinal components of our study are sufficient to detect meaningful effects with an acceptable level of statistical power. By ensuring adequate sample sizes, we enhance the reliability and generalizability of our findings.

### 2.3 Missing data

For participants who dropped out between Time 1 and 2 in the second set of surveys, we performed independent sample *t*-tests to compare the major variables of the respective groups. The results revealed no statistically significant difference between the two groups (*p* = 0.16–0.88). Therefore, it could be assumed that these missing data occurred randomly.

### 2.4 Measurement method

#### 2.4.1 Goal self-concordance

Goal self-concordance was measured following Sheldon and Elliot’s (1999) established procedure. The participants noted their primary recent goal, rating it across four motivational dimensions: external, introjected, identified, and intrinsic motivations. The first two and last two dimensions represent controlled and autonomous motivations, respectively. The scales were originally developed in English and thereafter translated into Chinese through back-translation validation. The original questionnaire was translated into Chinese by two graduate students of psychology, and the scale was back-translated by two graduate students of English majors. The differences were discussed after comparison and submitted to a professor of the Department of Psychology for review and proofreading, and the Chinese version of the scale was finally approved. A sample item is “I choose this goal because of pressure from others or circumstances.” A seven-point Likert scale, from 1 (completely disagree) to 7 (completely agree), was used. Consistent with prior studies, the goal self-concordance score was determined by subtracting the average of controlled motivations from that of autonomous motivations (Sheldon et al., 2004). In the three measurements of the sample in this study, the Cronbach’s α coefficients of this scale were 0.73, 0.70, and 0.71, respectively.

#### 2.4.2 Ego identity status

This study used the Ego Identity Status Scale developed by Kato (1983) and subsequently translated and adapted by Zhang (2000) to assess participants’ ego identity. The scale comprises three subscales—present commitment, past crisis, and future commitment pursuit—totaling 12 items labeled from A to L. A sample item is “I am working hard to achieve my goal.” Responses for each item were gaged on a six-point scale, ranging from “strongly disagree” to “strongly agree.” The scoring method for the subscales involved calculating the present commitment score as A-B + C-D + 14; the past crisis score as H-G + F-E+14; and the future commitment pursuit score as I-J + K-L + 14. These scores were integrated through a specific flowchart to determine six ego identity statuses, ranked from high to low developmental levels: 6 (achievement, A), 5 (foreclosure-achievement intermediate, A-F), 4 (foreclosure, F), 3 (moratorium, M), 2 (diffusion-moratorium intermediate, D-M), and 1 (diffusion, D). Higher scores signified more advanced development in ego identity. In the study’s initial sample, the scale’s Cronbach’s alpha coefficient was 0.72, while in the subsequent two surveys of the second sample set; the coefficients were 0.73 and 0.70.

## 3 Results

### 3.1 Common-method variance test

Given that data were collected via self-report methods, a potential CMV risk arose. To address this, the study followed Zhou and Long’s (2004) guidelines in implementing control measures during testing. These measures included ensuring participant anonymity, reaffirming that data would be exclusively utilized for scientific purposes, using scales with reverse-scored items, and adding attention-check questions within the survey. Furthermore, the distinct sample groups for the two survey sets, coupled with the longitudinal design of the second set involving data collection at two separate time points, served to mitigate the risk of CMV. Additionally, Harman’s single-factor test was conducted for statistical control prior to data analysis, entailing an unrotated principal component factor analysis of all survey items. The analysis identified nine factors with eigenvalues exceeding 1. The largest factor explained 19.38% of the total variance, with the remaining factors under the 40% threshold, thus indicating no significant presence of CMV in the data.

### 3.2 Descriptive statistics and correlational analysis

Table 1 presents the means, standard deviations, and correlations among the main variables and their dimensions based on the first set of survey data (*N* = 292). The data reveal that goal self-concordance positively correlates with ego identity status, present commitment, and future commitment pursuit (*r* = 0.34, 0.38, 0.41, *p* < 0.01). Similarly, autonomous motivation shows positive correlations with ego identity status, present commitment, and future commitment pursuit (*r* = 0.32, 0.33, 0.38, *p* < 0.01). Conversely, controlled motivation negatively correlates with these same variables (*r* = −0.23, −0.29, −0.30, *p* < 0.01).

**Table 1:** Descriptive statistics and correlations of variables: first set of survey data (*N* = 292).

	M	SD	1	2	3	4	5	6
1. Goal self-concordance	1.16	2.18						
2. Autonomous motivation	5.23	1.16	0.74^**^					
3. Controlled motivation	4.07	1.53	−0.86^**^	−0.30^**^				
4. Ego-identity status	2.96	1.44	0.34^**^	0.32^**^	−0.23^**^			
5. Past crises	16.40	2.65	0.11	0.10	−0.09	0.25^**^		
6. Present commitment	17.46	3.40	0.38^**^	0.33^**^	−0.29^**^	0.82^**^	0.20^**^	
7. Future commitment pursuit	17.47	2.96	0.41^**^	0.38^**^	−0.30^**^	0.59^**^	0.30^**^	0.64^**^

Additionally, we calculated the coefficients of determination (*R*^2^) to quantify the proportion of variance in goal self-concordance explained by ego identity status. The corresponding *R*^2^ value is 0.12, suggesting that approximately 12% of the variance in goal self-concordance can be attributed to ego identity status. This relatively small coefficient of determination implies the contribution of other factors to variations in goal self-concordance.

Table 2 details the means, standard deviations, and correlations for the second set of survey data (*N* = 98). It demonstrates a positive correlation between Time 1 ego identity status and Time 1 goal self-concordance (*r* = 0.23, *p* < 0.01), and between Time 2 ego identity status and Time 2 goal self-concordance (*r* = 0.31, *p* < 0.01), consistent with the findings from the first survey. This consistency underscores the stability of the positive correlation between ego identity status and goal self-concordance.

**Table 2:** Descriptive statistics and correlations of variables: second set of survey data (*N* = 98).

	M	SD	1	2	3
1. Time 1 ego identity status	2.99	1.39			
2. Time 1 goal self-concordance	1.33	1.91	0.23^*^		
3. Time 2 ego identity status	2.74	1.33	0.53^**^	0.19	
4. Time 2 goal self-concordance	1.39	1.88	0.30^**^	0.44^**^	0.31^**^

Additionally, a positive correlation exists between Time 1 ego identity status and Time 2 goal self-concordance (*r* = 0.30, *p* < 0.01); however, the relationship between Time 1 goal self-concordance and Time 2 ego identity status is not statistically significant (*p* > 0.05).

Furthermore, both Time 1 ego identity status and Time 1 goal self-concordance exhibit positive correlations with their respective Time 2 measures (*r* = 0.53 for ego identity status, *r* = 0.44 for goal self-concordance; *p* < 0.01), suggesting inherent stability in these constructs without intervention. Therefore, Hypothesis 1 was supported.

### 3.3 Analysis of variance

**Figure 1:**
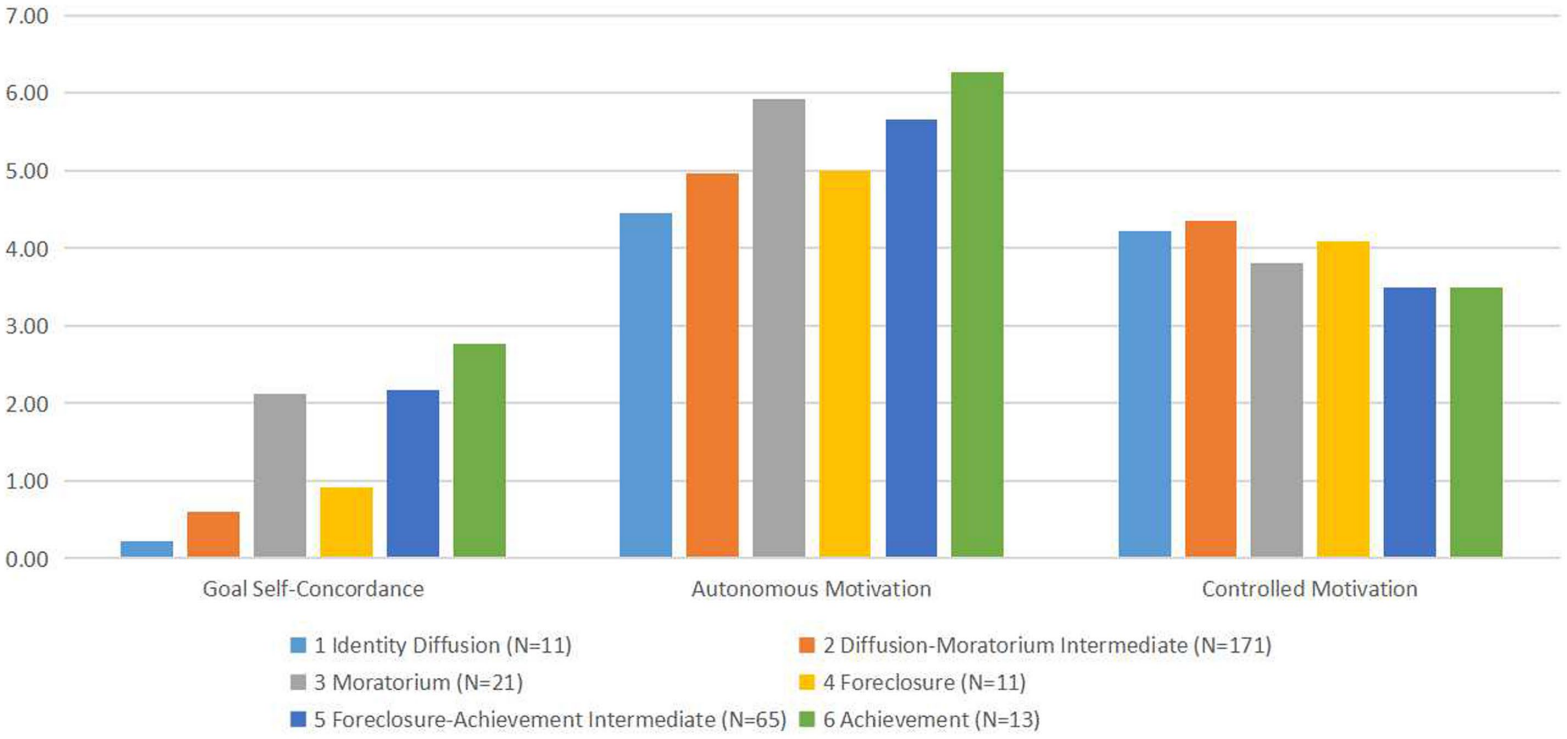
Analysis of variance plots for main variables across different ego identity statuses.

An ANOVA was conducted on the first survey data to examine the effects of different ego identity status on goal self-concordance and its dimensions. Ego identity status type served as the grouping variable, with goal self-concordance, autonomous motivation, and controlled motivation as dependent variables. The analysis, as shown in Table 3 and Figure 1, revealed significant differences across the six ego identity categories.

**Table 3:** ANOVA of variables across different ego identity statuses (M ± SD).

	Goal self-concordance	Autonomous motivation	Controlled motivation
1. Identity diffusion (*N* = 11)	0.23 ± 2.83	4.46 ± 1.52	4.23 ± 1.59
2. Diffusion-moratorium intermediate (*N* = 171)	0.61 ± 1.98	4.96 ± 1.06	4.35 ± 1.41
3. Moratorium (*N* = 21)	2.12 ± 1.86	5.93 ± 0.64	3.81 ± 1.65
4. Foreclosure (*N* = 11)	0.91 ± 2.61	5.00 ± 1.22	4.09 ± 1.80
5. Foreclosure-achievement intermediate (*N* = 65)	2.17 ± 2.10	5.66 ± 1.23	3.49 ± 1.58
6. Achievement (*N* = 13)	2.77 ± 1.73	6.27 ± 0.70	3.50 ± 1.67
*F*	8.62	9.49	3.67
*p*	0.000	0.000	0.003

### 3.4 Cross-lagged analysis

**Figure 2:**
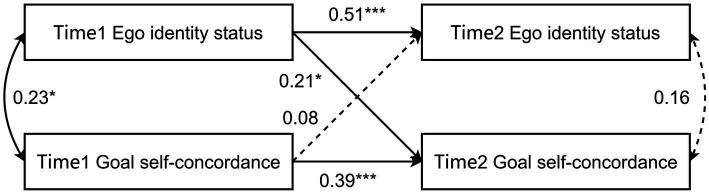
Results of the cross-lagged analysis for ego identity status and goal self-concordance.

To further examine the causal relationship between ego identity status and goal self-concordance, a cross-lagged analysis was performed on the longitudinal data from the second survey set. This analysis entailed constructing a cross-lagged model based on the survey results of ego identity status and goal self-concordance at two distinct time points. The model achieved saturation [root mean square error of approximation (RMSEA) = 0; standardized root mean square residual (SRMR) = 0; comparative fit index (CFI) = 1; Tucker Lewis Index (TLI) = 1], with detailed results depicted in Figure 2.

The stability paths for both ego identity status (β = 0.51, *p* < 0.001) and goal self-concordance (β = 0.39, *p* < 0.001) were significant, demonstrating that both constructs are relatively stable over time. The cross-lagged path from Ego Identity Status at Time 1 to goal self-concordance at Time 2 was significant (β = 0.21, SE = 0.09, *t* = 2.34, *p* < 0.05), suggesting that higher ego identity status at Time 1 predicts higher goal self-concordance at Time 2. The cross-lagged path from goal self-concordance at Time 1 to ego identity status at Time 2 was not significant (β = 0.08, SE = 0.09, *t* = 0.90, *p* > 0.05), indicating that Time 1 goal self-concordance did not significantly predict Time 2 ego identity status. Therefore, Hypothesis 3 was supported.

## 4 Discussion

### 4.1 Relationship between ego identity status and goal self-concordance

The correlation analyses revealed a significant positive relationship between ego identity status and goal self-concordance. However, the coefficient of determination (*R*^2^ = 0.12) indicates that ego identity status explains only 12% of the variance in goal self-concordance. This suggests that other factors, such as personal values, intrinsic motivation, and environmental influences, likely play a significant role in determining goal self-concordance.

When focusing on the three dimensions of ego identity status, past crisis did not exhibit a significant relationship with either goal self-concordance, autonomous motivation, or controlled motivation. By contrast, both present and future commitment pursuit demonstrated strong positive correlations with goal self-concordance and autonomous motivation, and equally strong negative correlations with controlled motivation. This pattern indicates that present and future self-commitment significantly and positively influence goal self-concordance, whereas past crisis exerts no notable effect.

The reason for this could be that past crises assess whether individuals have faced uncertainty and confusion over pivotal issues in their growth, such as personal beliefs, career plans, and social roles (Kato, 1983; Huang et al., 2012). The influence of a past crisis on goal self-concordance is not solely based on the crisis itself but is more dependent on the overall state of ego identity status development. If an individual has experienced a substantial past crisis and has reached a high level of present commitment, resulting in a state of identity achievement, then the impact on goal self-concordance is positive. Conversely, if an individual has undergone a significant past crisis but remains in a state of confusion or contemplation, in either an identity moratorium or diffusion status, the effect on goal self-concordance turns negative. Furthermore, goal self-concordance appears to be largely influenced by an individual’s current state. Those who actively engage in self-exploration presently and aspire for further self-exploration tend to show enhanced awareness and valuation of their internal interests and values. Consequently, they are more inclined to set goals with higher levels of self-concordance.

### 4.2 Differences in goal self-concordance across various ego identity status

This study delved into the relationship between various levels of ego identity status and goal self-concordance, including its dimensions, and uncovered significant differences across these statuses. Pairwise comparisons revealed distinct impacts on goal self-concordance: identity-achievement status ranked highest, followed by achievement-foreclosure intermediate, moratorium, foreclosure, and moratorium-diffusion intermediate statuses, with diffusion status being the lowest. Notably, the mean values for identity achievement, achievement-foreclosure intermediate, and moratorium were significantly higher than those for foreclosure, moratorium-diffusion intermediate, and diffusion. For autonomous motivation, the order was identity achievement > moratorium > achievement-foreclosure intermediate > foreclosure > moratorium-diffusion intermediate > diffusion; and for controlled motivation, it was moratorium-diffusion intermediate > diffusion > foreclosure > moratorium > identity achievement > achievement-foreclosure intermediate.

Interestingly, although Kato (1983) ranked moratorium slightly lower than foreclosure in ego identity status developmental levels, this study found its goal self-concordance level to be significantly higher than that of the foreclosure status. This discrepancy may relate to the uniqueness of the university students’ demographics. University years are a time of acceptable delay in ego identity establishment; even if students have not achieved identity-achievement status, moratorium signifies a higher future commitment pursuit. Foreclosure may increase controlled motivation and decrease autonomous motivation; thus, students in moratorium have higher goal self-concordance than those in foreclosure status.

### 4.3 Reciprocal predictive relationship between ego identity status and goal self-concordance

This study investigated the reciprocal predictive relationship between ego identity status and goal self-concordance using a correlational analysis of cross-sectional data and cross-lagged analysis of longitudinal data.

The correlational analysis of both datasets at two time points consistently demonstrated a significant positive correlation between ego identity status and goal self-concordance, underscoring the stability of this relationship. Moreover, the cross-lagged analysis of the follow-up data indicated that Time 1 ego identity status significantly predicted Time 2 goal self-concordance. This finding is consistent with Schwartz et al.’s (2005) perspective that ego identity serves as a life course anchor, offering foundational support and direction for goal formation. It also corroborates Burrow and Hill’s (2020) assertion that ego identity status is key to the development of a life purpose, with successful navigation through ego identity status crises fostering new strengths and abilities, thereby enhancing goal exploration and achievement.

However, the analysis revealed that Time 1 goal self-concordance did not predict Time 2 ego identity status. This implies that while a significant correlation exists between ego identity status and goal self-concordance in cross-sectional studies, the relationship is not bidirectional. In this dynamic, ego identity status functions as the cause and goal self-concordance serves as the effect. This means that although people may set self-consistent goals for themselves at one point in time, this does not mean that they have a higher level of ego identity status. However, a higher level of ego identity status can cause people to steadily obtain higher goal self-concordance.

### 4.4 Limitations and future research directions

Although this study contributes to a clearer understanding of the relationship between ego identity status and goal self-concordance, some limitations remain. First, the data were collected through online surveys, which better ensures anonymity but is more likely to lead to sample loss. Therefore, the effective sample size obtained in the longitudinal study is small. However, we also ensured the validity of the data analysis by examining missing data and statistical test force analysis. Second, cultural factors play a significant role in shaping ego identity and goal self-concordance. For instance, collectivist cultures may emphasize community-oriented goals, while individualist cultures prioritize personal ambitions. These cultural nuances can influence the strength and nature of the relationship between ego identity and goal self-concordance. In this study, only Chinese college students were selected. Therefore, the results of the study will be more applicable in collectivist countries. It is important to recruit participants from other cultures to enhance the generalizability of the study.

Future research could further explore the mechanisms underlying the reciprocal relationship between ego identity status and goal self-concordance, considering cultural variations. Longitudinal studies with larger and more culturally diverse samples could provide deeper insights into how these constructs interact over longer periods and across different cultural contexts. Additionally, examining the role of external factors such as social support, cultural influences, and life transitions could offer a more comprehensive understanding of the dynamics between identity formation and goal setting.

## 5 Conclusion

This longitudinal study has provided important insights into the relationship between ego identity and goal self-concordance among university students. Our findings underscore the critical role of a well-developed sense of identity in the process of goal setting, highlighting several key conclusions that align with the existing literature.

Firstly, our results demonstrate a significant positive correlation between ego identity status and goal self-concordance. Students who exhibit higher levels of current and future identity commitment tend to set goals that are more aligned with their intrinsic values and interests. This suggests that the development of a strong and coherent ego identity is essential for effective and meaningful goal setting.

Additionally, we observed notable differences across identity statuses. Students in the identity achievement status reported the highest levels of goal self-concordance, followed by those in the moratorium and foreclosure statuses, while those in the diffusion status exhibited the lowest levels. These findings emphasize the importance of fostering identity achievement to enhance goal self-concordance.

Importantly, our study also reveals a causal relationship between ego identity status and goal self-concordance: ego identity status is the cause, and goal self-concordance is the effect. This highlights the foundational role of identity development in shaping goal-setting behaviors, demonstrating that a well-established ego identity leads to more self-concordant goals.

From a practical perspective, our findings suggest that educational and developmental interventions aimed at supporting identity development and commitment could lead to more self-concordant goal setting. Such programs could ultimately promote greater well-being and personal growth among students.

In summary, this study extends the implications of the goal self-concordance model, addressing why we might choose goals that potentially lead to unhappiness—because we have not yet developed a well-established ego identity. Answering this question provides insights into resolving the dilemma of cognitive and behavioral dissonance that people face when setting goals. People can increase their level of ego identity by increasing their present and future self-engagement, which will help them set goals that are more aligned with themselves.

## Data availability statement

The data supporting this study will be made available upon reasonable request to the corresponding author.

## Ethics statement

The studies involving humans were approved by Ethics Committee of Zhejiang University. The studies were conducted in accordance with the local legislation and institutional requirements. The participants provided their written informed consent to participate in this study.

## Author contributions

LC: Data curation, Investigation, Methodology, Writing – original draft. JM: Writing – review & editing.
